# Work–Life Balance? It Is Not about Balance, but Priorities

**DOI:** 10.3389/fped.2016.00006

**Published:** 2016-02-11

**Authors:** Keiko M. Tarquinio

**Affiliations:** ^1^Department of Pediatrics, Division of Critical Care Medicine, Emory University, Atlanta, GA, USA

**Keywords:** work–life balance, critical care medicine, research, career, women

Work–life balance is a never ending topic of discussion among physicians. We constantly discuss how to juggle career advancement, marriage, and children in our daily professional life. Work always seems to be antagonistic to, rather than synergistic with, maintaining a personal life. However, true balance does not sink in until a moment arrives in your life when you consider giving up your career.

After finishing my critical care fellowship, things seemed to be going fairly well in my life. I had my family, my house, my job as a critical care physician, and my passion to do research. I love my work and am truly honored by the privilege to work with critically ill children and their families, who need good clinical advice and help to make the best decision for their precious ones. Yes, there are difficult moments physically and mentally for myself and my staff, but the fact that I can contribute to someone’s critical life decisions has always motivated me and kept me going.

Despite being discouraged from pursuing a career as a physician scientist, I was stubborn enough to work hard and to obtain grants to launch my research projects. I was warned that I would have little success in balancing my life between my family, job as a critical care physician and role as a researcher. However, I enjoyed every painful, fun moment writing manuscripts and IRB applications and tried to collaborate with researchers from multiple disciplines. My view of medicine became wider and richer through research. When I met successful female physician scientists through the K 12 program and the Association of American Medical Colleges (AAMC) meeting, I was especially energized and the discouraging words against pursuing a career as a physician scientist faded away. Knowing that everybody goes through the same stage of life (struggling with career, family, and/or research) made me think that I am not alone in feeling overwhelmed and undervalued.

During weekdays, I did not mind working long hours as a single mother with my daughter while my husband was in training 1500 miles away. We only spent precious time as a united family during the weekend and went back to work doing research or being on service and taking calls in the ICU every Monday night. I still worked at night after my daughter went to sleep and took advantage of my husband being with my daughter during weekends.

When my daughter was about 20 months old, my husband became critically ill (without knowing the long-term sequelae), my life stability suddenly became very fragile without warning. I was pregnant with my second child, and everything became paralyzed. It is the worst experience for anyone to have your family member admitted to the ICU, but worse to know about everything that is happening around you, and unable to hide the fact that you are an intensivist. I went through so many scenarios without answers in my head and realized that time is the only medicine to take care of this crisis. What if he was neurologically devastated? Would he come home with a tracheostomy and mechanical ventilation? Would I have any help to take care of my husband and children? Would my children ever play with their daddy? During his hospitalization, I completely shut down any in-coming contacts, including flower delivery, family visits, and phone calls. I wanted to be a wife next to my husband who was paralyzed and mechanically ventilated, not an intensivist. All my colleagues were wonderful helping me to get through this crisis, and I learned a lesson from them – unless I was well, I could not take care of my husband, daughter, or unborn child.

After this extreme situation, the importance of my family as a foundation became clear, and being a physician or a researcher is now an additional factor to enrich my life. Right after discharge from the ICU, a 2013 nor’easter covered the New England area in snow. My husband insisted that we go skiing 2 days after discharge from the ICU. Now, I switched to my role as an intensivist to tell him “no”; however, my wish was defeated by his strong desire. Ski boots helped his foot drop, and albuterol in my pocket gave me a sense of security. However, seeing my husband standing on the snow-covered slope was the most effective spiritual therapy for both of us to recover from the delirium and posttraumatic stress disorder brought on by the ICU. I never had so much appreciation for having a simple but complete life. Sipping a cup of apple cider in our hands and joking with my husband with smiles on our faces, the snow-covered mountain reminded me of what is most important in life: family.

Several studies have been done by groups, including the AAMC looking at female physicians’ career success. Despite the increase in the number of women entering medical school and residency over the years (close to 50%), only one-third of full time faculty and 21% of full professors are women (1). The progress and challenges for women in academic medicine are not new (2). Little flexibility for promotion criteria and a narrowly defined “success” path in academia that is more suitable for males does not accommodate the differences in women’s career trajectories (2). At the same time, one study showed that female physicians with children have lower value in terms of career success and career support experiences than male physicians. In the same study, females showed higher levels of life satisfaction overall, where friends, leisure activities, and income are concerned, despite the negative impact that parenthood has on these career factors (3). Where do I stand now?

In my case, balance is based on the pyramid of my family at the bottom and work at the top, instead of parallel to each other. Neither should conflict with the other. Being an intensivist, my appreciation of life sank in even deeper after my learning lesson. I cannot sustain myself without this foundation. From these critical moments, I decided to devote 100% of my time to family when I am not in the hospital.

In one survey looking at work–life balance, both male and female gynecologists reported that work–life affected their private life in a similar way, causing them to neglect both their partner and children due to their work (4). I cannot neglect my family, even when I am at work, and I should not. There are many intensivists available to take care of sick children’s; however, I am the only one for my family. I try not to stay up late to write grants and manuscripts at home, and I do not feel guilty about it. Instead, I spend my time cooking for my family, bathing, and reading books to my children daily. I make their bento box and drop them off at school as long as I am not on service. This is part of my life as a mommy intensivist. If I did not have children, I would have spent the time on career development, including educating residents/fellows, and administrations. I am probably juggling between work and kids in a similar way to other scientists in the laboratory (5). However, as an intensivist dealing with a critical situation at the bedside, I cannot leave my patient to come back the next day the way that a basic scientist can. If I had to leave the lab I could always repeat any experiments that failed in my absence, but I cannot apply the same mentality to my patients in the intensive care unit. This is how my clinical and research work get prioritized. Prior having my children, I chose to devote my time on research. I may not be a successful world-class researcher, but I am persistent with what I feel is right at the moment, and I feel good about doing something I am passionate about. I may prioritize my piles of work terribly some days, and I may do fairly well other days. That is okay with me. I have to accept my abilities and myself at my level of career and family requirements.

Sometimes, I may have to say no to my colleagues based on my priorities. I ask my help from my colleagues when I need, and I offer my help to others when I can. My family foundation gives me stability, energy, and appreciation at work. I look at the world through very different glasses than before I was a parent. The goal and purpose of life can be liquid, and I embrace them at each stage of my life. My priority has changed over years as my training and career have advanced, and I personally think that is how life should be. One may have issues with own parents or siblings even one may not be married and not having children, and taking care of them as a priority over the career. When my children grow up and do not need me right next to them, I will shift my priorities to what motivates me at that time.

Many factors contribute to women physicians leaving academic institutions early in their career (6). However, Levine et al. concluded that disconnection or discrepancy between their own priorities and those of the institution is the main reason for women physicians to leave academic institutions early in their career. I personally think improvement in work–life balance comes from having a conducive work atmosphere and a customized approach to individual needs on an institutional level of understanding and support.

I would encourage connecting yourself with peers in similar situations in your own institution. Sometimes, human resources or administration can facilitate this through faculty development courses or seminars; these may provide networking opportunities among faculty with similar career goals. I attended as many of these as I could to learn from the others with different perspectives how to overcome difficult situations. At the national level, the AAMC is a great organization to provide mentoring, networking, and opportunities to know peers through, for example, the Early Career Women Faculty Professional Development Seminar. As Sapey concluded, we may see light coming through the old school system, evolving to become better suited to physicians with families (7). One way or another, everyone goes through similar stages of life or crisis. So, why do not we respect each other and support our journey toward success?

At the end of my life, I would like to be recognized as a wife and a mother first, rather than a physician or researcher in a particular field. This is my priority.

## Author Contributions

KMT was the sole author. No other contributors to this article.

## Conflict of Interest Statement

The author declares that the research was conducted in the absence of any commercial or financial relationships that could be construed as a potential conflict of interest.
